# Effects of Salinity Fluctuation on Antimicrobial Resistance and Virulence Factor Genes of Low and High Nucleic Acid-Content Bacteria in a Marine Environment

**DOI:** 10.3390/microorganisms13071710

**Published:** 2025-07-21

**Authors:** Wei Hu, Xinzhu Zhou, Yu Liu, Yadi Zhang, Yingying Wang

**Affiliations:** 1 College of Tobacco Science and Engineering, Zhengzhou University of Light Industry, Zhengzhou 450001, China; hvvhuwei@126.com; 2 Key Laboratory of Pollution Processes and Environmental Criteria (Ministry of Education), Tianjin Key Laboratory of Environmental Remediation and Pollution Control, Nankai International Advanced Research Institute (Shenzhen Futian), College of Environmental Science and Engineering, Nankai University, Tianjin 300350, China; zhouxinzhu321@163.com (X.Z.); nkuliuyu@163.com (Y.L.); zhangyadi@mail.nankai.edu.cn (Y.Z.)

**Keywords:** salinity, HNA bacteria, LNA bacteria, ARGs, VFGs

## Abstract

Salinity, as one of the critical environmental factors in marine ecosystems, has complex and wide-ranging biological effects. However, the effects of salinity fluctuation on antibiotic resistance genes (ARGs) and virulence factor genes (VFGs) in the marine environment are not well understood. In this study, metagenomic sequencing analysis was used to reveal the response of ARGs and VFGs, hosted by low and high nucleic acid-content bacteria (HNA and LNA bacteria), to salinity, as it decreased from 26‰ to 16‰. The results showed that a total of 27 ARG types and 13 VFG types in HNA and LNA bacteria were found. Salinity changes had significant effects on the ARGs’ and VFGs’ composition and their hosts’ composition. In the network topology relationship, the complexity of the network between the ARGs and their host as well as the VFGs and their host differed with the decrease in salinity. The abundance of most genera of HNA and LNA bacteria was significantly corrected with the abundance of ARGs and VFGs, respectively. Overall, this study demonstrates the effects of salinity on ARGs and VFGs hosted by HNA and LNA bacteria in the marine environment and suggests the importance of salinity in regulating HNA and LNA bacterial communities and functions.

## 1. Introduction

Antibiotics have made outstanding contributions to eliminating human diseases over the last nearly one hundred years [1]. However, antibiotic resistance genes (ARGs) caused by their abuse have become a new type of pollutant and pose a major threat to global public health and ecological health [2]. ARGs exhibit broad dissemination through diverse pathways, including hospitals, sewage, animal waste, soil, and oceans [3]. Aquatic ecosystems are of the highest concern among the natural environments [4]. Previous studies have shown that ARGs are very widespread and diverse in the marine environment [5]. ARGs show great diversity in bacteria, and different bacterial species may carry different ARGs. Alarmingly, these ARGs can transmit not only among bacteria but also among humans, animals and the environment [2,6,7]. In addition to ARGs, the presence of virulence factor genes (VFGs) enables the bacterial host to invade humans or animals and cause disease [2]. The pathogenicity of bacteria is related to the VFGs in its genetic material and is jointly determined by the synergistic action of multiple virulence factors [8]. When ARGs and VFGs coexist in the genome of bacteria, the bacteria can not only evade antibiotic treatment, but also cause diseases and increase the risk to human health. VFGs and ARGs can spread among microorganisms, resulting in their wide distribution in the natural environment [7]. Recent advances in metagenomics-based studies have revealed various ARGs and VFGs in the Arctic permafrost region [2], the plastisphere [9], algae-associated bacteria [10], Tibetan glaciers [6], drinking water [11], estuaries [12] and the marine environment [3].

Based on detection by flow cytometry (FCM), it was found that the characteristics of bacteria were linked with the parameters in FCM, such as low fluorescence linked to low DNA content, low scatter linked to small cell size, filterability linked the small cell size, small cell sizes linked to small genome sizes and low DNA content [13,14,15,16]. Thus, the bacteria in the environment can originally be divided into two taxonomic groups, of low nucleic acid-content (LNA) bacteria and high nucleic acid-content (HNA) bacteria [17,18,19,20,21,22]. Researchers have investigated the abundance, activity, community composition and ecological functions of HNA and LNA bacteria in marine and freshwater environments [19,21,23,24]. Previous studies have reported that pathogenic bacteria exist in the composition of the LNA bacterial community, such as *Pseudomonas alcaligenes*, *Pseudomonas aeruginosa* and *Mycobacterium gordonae* [25], and the pathogenic genomic islands in the marine environment, and most of the genes are related to antibiotic resistance [26]. LNA bacteria, with their small cell size, can enter human cells and even the cell nucleus and play a key role in the pathogenesis of human diseases (such as the cause of cancer) [27]. For example, the Candidate phylum TM7 (with a genome of approximately 0.7 Mbp), a representative strain of typical LNA bacteria, is associated with the occurrence of human inflammatory mucosal diseases [28]. Furthermore, in the metabolic pathways of LNA bacteria in freshwater environments, there are multiple metabolic pathways related to human diseases, such as infectious diseases, bacterial diseases, immune diseases, etc., [29].

Alterations in environmental factors (such as salt concentrations, nutrient status and pH) can impose selection pressures on the evolution of bacterial communities, which are also linked to the emergence of antibiotic resistance [30]. The distribution of VFGs and ARGs in these environments is driven by a combination of abiotic factors [9,31], biological factors and spatial factors [2]. A previous study showed that environmental gradients could greatly affect the distribution patterns of ARGs and bacterial communities [32]. As one of the major environmental factors in the marine environment, salinity has been reported to have a significant effect on the community composition of marine microorganisms [23,24,33]. Marine bacteria are possible reservoirs of ARGs [34] and the distribution of ARGs in oceans have been reported in previous studies [3,35,36]. Our previous study indicated that salinity changes have a significant effect on the diversity and ecological functions of HNA and LNA bacteria [23], but empirical evidence of the effect of salinity on ARGs and VFGs in HNA and LNA bacteria in the marine environment is lacking.

This work reports the types and abundance of ARGs and VFGs in HNA and LNA bacterial communities in seawater. The aims of the present study were to investigate the ARGs and VFGs in sampled seawater by examining: (i) variations in the types of ARGs and VFGs in HNA and LNA bacterial communities in seawater across different salinities; (ii) the abundance of ARGS and VFGs and elucidate their dynamic response to salinity; and (iii) to identify the HNA and LNA bacterial hosts of ARGs and VFGs and elucidate the dynamic patterns of HNA and LNA bacterial hosts with salinity changes.

## 2. Materials and Methods

### 2.1. Sample Collection and Physicochemical Parameters

Some 300 L of water samples were taken from the Bohai Sea in China in May 2022, and stored at 4 °C during transportation. A Portable Thermol Multifunctional Meter (ORION 520M-01A, Thermo Fisher Scientific, Waltham, MA, USA) was used for in situ measurement of the physicochemical parameters (water temperature (T), pH and salinity). The high-temperature (680 °C) catalytic oxidation method on a TOC analyzer (multiN/C3100, Analytikjena, Jena, Germany) was utilized to measure the total organic carbon (TOC), total carbon (TC) and total nitrogen (TN). Total phosphorus (TP) was measured using a multiparameter water quality analyzer (DR3900, Hach Company, Loveland, CO, USA).

### 2.2. Flow Cytometry, Fluorescent Staining and Filtration Protocol

Flow cytometry was used to measure the total cell concentration (TCC) in the water samples [24]. The specific experimental methods are as described in the literature [23]. In short, 0.5 mL of the sample was placed in 1.5 mL sterilized centrifuge tubes and incubated at 37 °C for 5 min. Subsequently, 5 μL of fluorescent staining agent was added to each sample. After continuing to incubate in the dark for 10 min, the samples were loaded for bacterial count detection. The threshold on the green fluorescence channel (FITC-A) was set at 1000 to exclude instrument noise. The water sample was successively passed through 0.45 μm and 0.1 μm filter membranes in order to collect HNA and LNA bacteria. The a 0.45 μm membrane filter (Durapore^®^, Merck Millipore, Burlington, MA, USA) was used to capture the HNA bacteria and a 0.1 μm (Durapore^®^, Merck Millipore, USA) membrane filter (Durapore^®^, Merck Millipore, USA) was used to capture the LNA bacteria from the 0.45 μm filtrate. Filtration volumes were adjusted so as to collect an approximately equal number of 10^8^ cells on the membrane filter [21].

### 2.3. Continuous Experimental Design

In this experiment, a biological continuous reactor was used to set up three salinity gradients (26‰, 21‰, and 16‰) for the purpose of exploring how continuous salinity changes affect bacterial populations in the marine environment in this experiment. The experiment was conducted in three stages (Figure S1).

Stage 1: At the beginning of the experiment, 18 L (with a salinity of 26‰) of the original seawater sample was injected into the bioreactor through a peristaltic pump. The same volume of samples was taken at the same time every day for bacterial concentration detection. When the bacterial concentration stabilized, the experiment at this stage was ended.

Stage 2: Sterile fresh water samples were injected into the bioreactor by peristaltic pumps to reduce the salinity from 26‰ to 21‰. Then fresh water injection was stopped in order to continue the cultivation. The changes in bacterial concentration were monitored at regular intervals every day. Once the bacterial concentration stabilized, this stage of the experiment was ended.

Stage 3: As with the treatment method in the second stage, sterile fresh water samples were injected into the bioreactors using peristaltic pumps to reduce the salinity from 21‰ to 16‰. Then, the injection of fresh water was stopped to continue the culture and monitor the changes in bacterial concentration.

At the end of each stage (Figure S2), samples were taken from the bioreactor for the isolation, enrichment and DNA extraction of HNA and LNA bacterial communities. Meanwhile, the physicochemical indicators were determined as shown in Table S1.

### 2.4. DNA Extraction, 16S rRNA Amplicon Sequencing, and Sequence Processing

The Power Water DNA isolation kit (QIAGEN GmbH, Hilden, Germany) was used for total DNA extraction from the original seawater samples as well as from the biological continuous reactor with different salinities. A Qubit^®^ 2.0 Fluorometer (Invitrogen, Thermo Fisher Scientific Inc., USA) was utilized to quantify the DNA yield. Metagenomic DNA was sequenced after library construction. In detail, metagenomic DNA was first fragmented into short pieces of approximately 350 bp. Subsequently, the ends of these DNA fragments were made complete and an A-tail was added. After that, when connected with the full-length splitter, metagenomic sequencing was carried out using the NEBNext^®^ ΜLtra™ sequencer (NEB, Ipswich, MA, USA). Once the metagenomic DNA process was finished, the original sequence was subjected to quality control by fastp software (https://github.com/OpenGene/fastp (accessed on 16 July 2025), version 0.20.0) to obtain valid data [37]. Then, the MEGAHIT software (https://github.com/voutcn/megahit (accessed on 16 July 2025), version 1.1.2) was utilized to assemble the clean reads of each sample separately, with the k-mer range from 21 to 141 stepped over, in order to produce the sample-derived assembly [38]. Genes were predicted based on the final assembly contigs (>500 bp) using MetaGeneMark (http://metagene.cb.k.u-tokyo.ac.jp/) [39]. The predicted genes ≥300 bp in length from all samples were pooled and combined based on ≥95% identity and 90% read coverage using CD-HIT [40]. Bowtie was utilized to realign the reads to predicted genes [41]. For the identification of possible ARGs and VFGs, DIAMOND BLASTP (v2) was used to search the representative sequences [42] against the Comprehensive Antibiotic Resistance Database (CARD, v3.1.2) [43] and Virulence Factor Database (VFDB, 2019) [44], respectively. DIAMOND BLASTP was also utilized to search these sequences against the NCBI non-redundant protein sequence database (NR) for taxonomic assignments [42]. To reduce the false-positive bias, more stringent thresholds (E-value < 1 × 10^−5^, identity >70%, and alignment length > 50) were used for the final annotation. Kallisto (v0.46.2) was utilized to calculate the TPM value of each sample [45]. A matrix table was formed by combining the DIAMOND BLAST results and the Kallisto results according to the “contig id”. The abundance for each annotation was calculated using the sum of TPM values along with the relevant ARGs or VFGs. The ARGs or VFGs were used for species annotation to identify the associated host bacteria [46]. Raw sequence datasets were uploaded to the NCBI Sequence Read Archive with the Bio-Project accession number PRJNA1103382.

### 2.5. Data Analysis

Statistical analyses were primarily conducted using R software (v4.3.2) [47]. Network diagrams were constructed with installation packages such as “igraph”, “Hmisc” and “qvalue” packages [48]. Principal component analysis (PCoA) was utilized to visualize the community composition of bacteria and genes using “vegan (version 2.0)” and “ggplot2 (version 2.2.0)” packages [24]. The Spearman correlation of two genes was computed, and based on this coefficient, effective genes for the network map construction were filtered. Finally, the network was visualized by Gephi (https://gephi.org) and topological features like each network node were computed. Partial mental test analysis was used to investigate the impacts of environmental factors on the composition of genes and their hosts [24]. Procrustes analysis was used to study the relationships between ARGs and VFGs, as well as the genes and their hosts [49].

## 3. Results

### 3.1. Prevalence of ARGs and VFGs in HNA and LNA Bacteria Revealed Using Metagenomics

**Figure 1:**
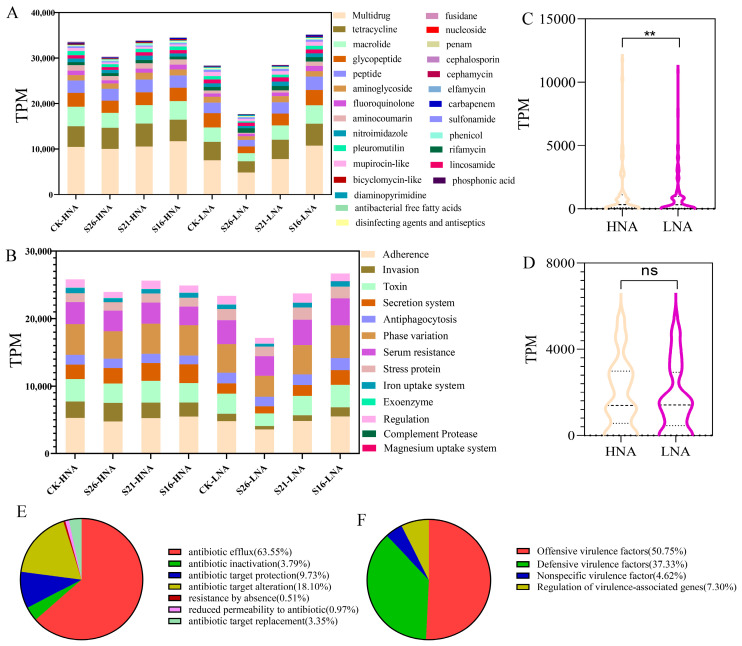
TPM values of ARGs (**A**) and VFGs (**B**) hosted by HNA and LNA bacteria in marine environment; statistical differences in ARGs (**C**) and VFGs (**D**) between HNA and LNA bacteria; type of ARGs (**E**) and VFGs (**F**). **: *p* < 0.01, ns: no significant.

In the present study, a total of 27 ARG types were detected in HNA and LNA bacteria with an average abundance of 1122.1 TPM (ranging from 0 to 11787.1 TPM) (Figure 1A). Among them, multidrug resistance genes and tetracycline resistance genes had the highest abundance in all samples, accounting for 15% and 7.2% of the total ARG abundance, respectively (Figure 1A). All the detected ARGs represented three major resistance mechanisms: antibiotic efflux (63.55%), antibiotic target alteration (18.10%) and antibiotic target protection (9.73%) (Figure 1E). Furthermore, most of the ARGs were more frequently encoded in chromosomes than in plasmids (Figure S3B). In addition, the co-occurrence of ARGs and MGEs was assessed, such as OprN-*tnp*A, *ole*C-IS91, *tet*A-IS91 in different samples (Figure S3C). This suggests that salinity change may lead to the horizontal transfer through plasmids.

A total of 13 VFG types were detected in HNA and LNA bacteria with the average abundance of 1839.3 TPM (ranging from 0 to 5571.5 TPM) (Figure 1B). Among them, adherence and invasion were represented with the highest abundance in all samples, accounting for 20.1% and 7.0% of the total VFGs abundance, respectively (Figure 1B). The detected VFGs represent two major types: offensive virulence factors (50.75%) and defensive virulence factors (37.33%) (Figure 1F).

The Mantel test and Procrustes analysis on the abundance of ARGs and VFGs revealed relatively strong correlation coefficients (*p* < 0.05) in HNA bacteria and LNA bacteria (Figure S4). Strikingly, the TPM values of the ARGs and VFGs in LNA bacteria showed an increasing trend with the decrease in salinity (Figure 1A,B). Here, the TPM values of ARGs in HNA bacteria were relatively significantly higher than those in LNA bacteria in all samples (Figure 1C) with no significant difference in the TPM value of VFGs between HNA bacteria and LNA bacteria (Figure 1D).

### 3.2. Effects of Decreased Salinity on the Abundance of ARGs and VFGs

**Figure 2:**
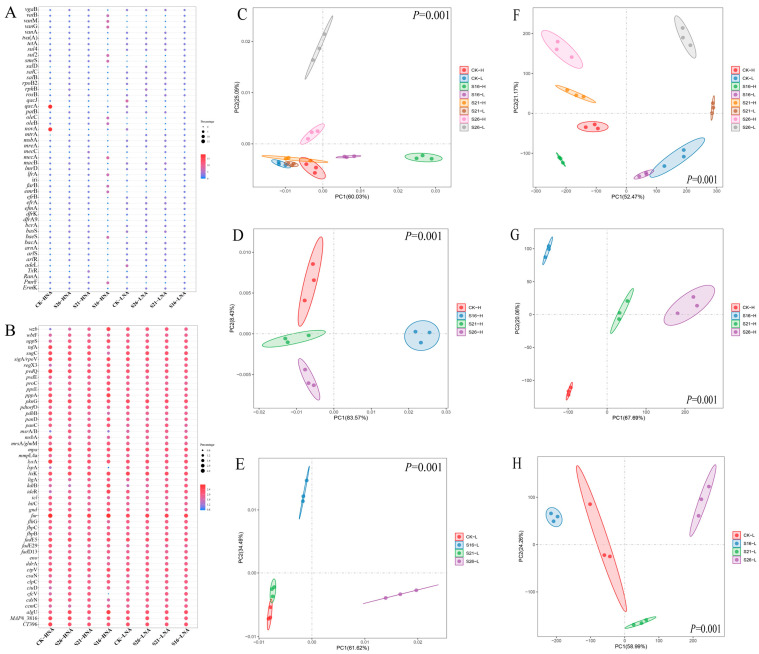
Bubble diagram of the top 50 most abundant ARGs (**A**) and VFGs (**B**). Principal coordinate analysis (PCoA) showing the distribution pattern of ARGs (**C**–**E**) and VFGs (**F**–**H**) in different salinities. CK-H: HNA bacteria in control group, CK-L: LNA bacteria in control group; S26-H: HNA bacteria in salinity of 26‰, S21-H: HNA bacteria in salinity of 21‰, S16-H: HNA bacteria in salinity of 16‰, S26-L: LNA bacteria in salinity of 26‰, S21-L: LNA bacteria in salinity of 21‰, S16-L: LNA bacteria in salinity of 16‰.

The most abundant ARGs (Top 50) identified in our results are shown in Figure 2A, which belonged to antibiotic efflux, antibiotic inactivation, antibiotic target alteration, antibiotic target protection and antibiotic target replacement mechanism (Figure S3A). As shown in Figure 2C, the ARG composition in HNA and LNA bacteria was notably distinct under the same salinity conditions, highlighting significant differences between the ARGs in HNA and LNA bacterial communities in the same salinity (*p* = 0.001). The ARG composition in LNA and HNA bacteria displayed significant separation across different salinity conditions, indicating substantial impacts from salinity on ARG composition in LNA and HNA bacteria (*p* = 0.001) (Figure 2D,E). These findings were further supported by partial Mantel analysis that the ARG composition in HNA and LNA bacterial communities were both significantly affected by changes in salinity (Table S2). The most abundant VFGs (Top 50) in our results are shown in Figure 2B. The results in the present study indicate that salinity changes may further affect the abundance of VFGs in the marine environment (*p* = 0.001) (Figure 2F–H). These findings were further supported by partial Mantel analysis that the VFG composition in the HNA and LNA bacteria were significantly strongly correlated to salinity changes (Table S3).

### 3.3. Co-Occurrence Network Analysis of ARGs and VFGs in the Bacteria

In order to investigate the potential interactions between ARGs and VFGs hosted by HNA and LNA bacteria, we mapped the structure of co-emerging genes (Figure S6). Spearman’s rank correlation coefficient between the genes was calculated and *p* < 0.001 and correlation coefficient r > 0.9 were selected to obtain the co-occurrence network of the genes. The node and link numbers of the co-occurrence network across ARGs and VFGs in both bacteria are shown in Table S4. Notably, the topological features of the network revealed that most of the nodes in the networks of ARGs and VFGs belonged to VFGs (LNA: 83.3%; HNA: 83.67%) (Figure S6A,B). In the present study, over half of the links in the co-occurrence network of ARGs and VFGs in both bacteria exhibited significantly positive correlations (HNA: 55.23%; LNA: 77.27%), respectively (Figure S6A,B), indicating that the relationship between ARGs and VFGs in both bacteria was predominantly coexistence.

Furthermore, the interactions of ARGs hosted by HNA and LNA bacteria were analyzed (Figure S6C). The topological features of the network are shown in Table S4. The results showed that most of the nodes in the networks of ARGs and VFGs hosted by HNA and LNA bacteria belonged to HNA bacteria (Figure S6C). This underscores the pivotal role of HNA bacteria in the network of ARGs hosted by the two groups. In this study, over half of the links in the co-occurrence network of ARGs exhibited significantly positive correlations (Figure S6C), indicating that the relationship of ARGs in both bacteria was predominantly coexistence, which was similar to the VFGs (Figure S6D).

### 3.4. Effects of Salinity on the Bacterial Community Hosting ARGs and VFGs in the Marine Environment

**Figure 3:**
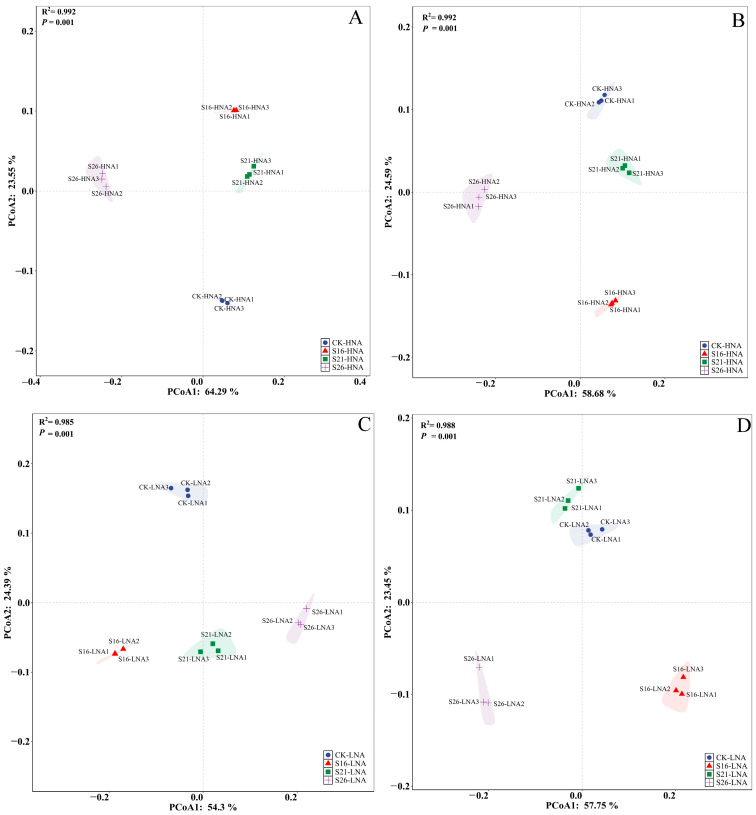
Schematic diagram of principal component analysis of HNA and LNA bacteria hosting the ARGs and VFGs. S26: 26‰, S21: 21‰, S16: 16‰; (**A**,**C**): ARGs; (**B**,**D**): VFGs.

**Figure 4:**
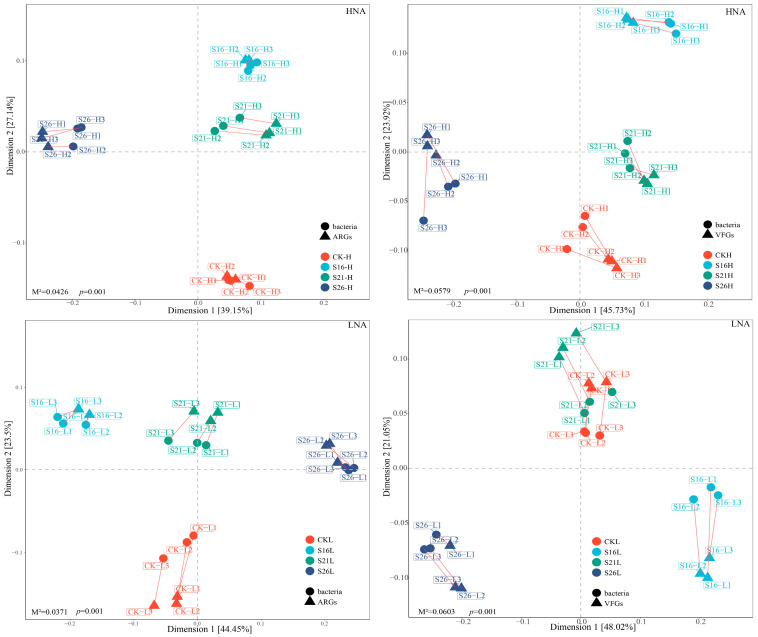
Procrustes analysis showing the significant correlations between bacterial community and ARGs, VFGs; S26H: HNA bacteria in salinity of 26‰, S21H: HNA bacteria in salinity of 21‰, S16H: HNA bacteria in salinity of 16‰, S26L: LNA bacteria in salinity of 26‰, S21L: LNA bacteria in salinity of 21‰, S16L: LNA bacteria in salinity of 16‰.

To identify the potential host bacteria for ARGs and VFGs, taxonomy annotation was conducted using the metagenomic data. The results revealed that Alphaproteobacteria and Gammaproteobacteria were the most abundant in HNA bacteria (Figure S7A,B). Alphaproteobacteria, Gammaproteobacteria and Actinobacteria were the most abundant classes in LNA bacteria (Figure S7C,D). *Maribita*, *Marinobacter* and *Spongiibacter* were the dominant genera in HNA bacteria (Figure S8A,B). Candidatus *Pelagibacter* and *Alcanivorax* were the dominant genera in LNA bacteria (Figure S8C,D). In the present study, both bacterial communities displayed significant separation across different salinity conditions, respectively (Figure 3). These findings were further supported by partial Mantel analysis (Tables S5 and S6). The changes in the composition of the HNA and LNA bacterial community were significantly consistent with those of ARGs and VFGs, respectively (Figure 4). Furthermore, pathogenic antibiotic-resistant bacteria (PARB), which carry both ARGs and VFGs, were analyzed (Figure S9). The ARG types of multidrug, tetracycline, macrolide, and VFGs associated with regulation, and toxins were more frequently identified in the PARB genera, mainly belonging to *Marinobacter*, *Pseudomonas*, *Vibrio*, *Alcanivorax*, and *Alteromonas* in HNA and LNA bacteria (Figure S9A,B). The results of further analysis showed that salinity changes had significantly affected PARB composition in HNA and LNA bacteria (*p* = 0.001) (Figure S9C,D).

**Figure 5:**
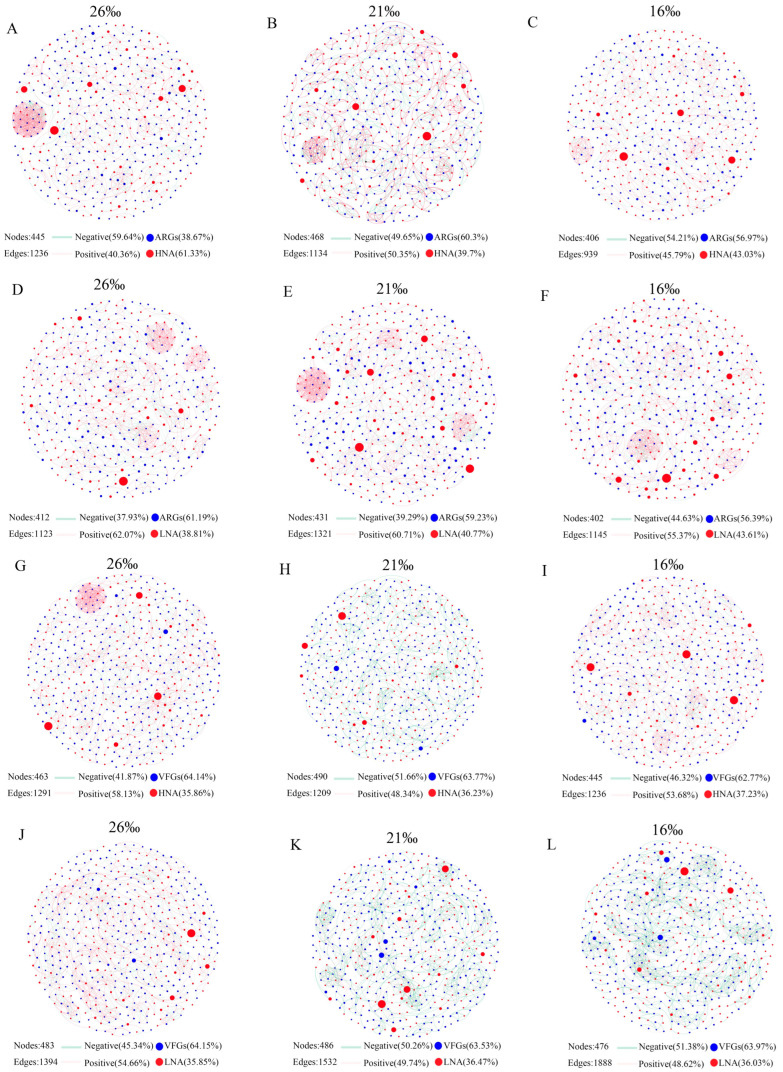
Network analysis revealing the co-occurrence pattern of ARGs, VFGs and core bacterial genera in different salinities with Spearman’s correlation coefficient r > |0.9| and *p* < 0.01. HNA: HNA bacteria; LNA: LNA bacteria. (**A**–**C**): the co-occurrence pattern of ARGs and HNA bacterial genera in different salinities; (**D**–**F**): the co-occurrence pattern of ARGs and LNA bacterial genera in different salinities; (**G**–**I**): the co-occurrence pattern of VFGs and HNA bacterial genera in different salinities; (**J**–**L**): the co-occurrence pattern of VFGs and LNA bacterial genera in different salinities.

To reveal the co-occurrence pattern of ARGs, VFGs and bacterial genera, the networks were visualized in different salinities based on Spearman’s rank correlation coefficient (with *p* < 0.01 and r > 0.9). Notably, the networks between the bacteria, ARGs and VFGs in different salinities exhibited unique co-occurrence patterns (Figure 5). The numbers of the nodes and edges in the network of HNA bacteria–ARGs, HNA bacteria–VFGs, LNA bacteria–ARGs, and LNA bacteria–VFGs vary under different salinity conditions (Figure 5; Table S4), indicating that the complexity of the network is affected by changes in salinity. In addition, in the present study, we found that the abundance of HNA and LNA bacteria hosting ARGs and VFGs had significant correlation with ARGs and VFGs, respectively (Figure S10). At the genus level, we further analyzed the relationship between the relative abundance of species and ARGs as well as VFGs. The results showed that the abundance of most genera of HNA and LNA bacteria was significantly correlated with ARGs and VFGs, respectively (Figure S11).

## 4. Discussion

The Bohai Bay is the largest semi-enclosed bay in the northeastern part of China. There are over 100 drainage channels along its coast, and 75% of these channels discharge pollutants, with origins that include fishery, animal husbandry, industry, agriculture, and so on, that exceed the acceptable limits [50]. The emission of these pollutants can intensify the accumulation of antibiotics in the marine environment, leading to the marine environment becoming a reservoir of ARGs [1,51,52]. In the present study, multidrug genes, tetracycline genes, macrolide genes, glycopeptide genes, peptide genes and so on were found in HNA and LNA bacteria (Figure 1A), which resembled the core resisters in other ecosystems, such as soil and freshwater systems [53]. And multidrug resistance genes and tetracycline resistance genes had the highest abundance in HNA and LNA bacteria (Figure 1A), which was similar to the previous study [3]. Another previous study reported that multidrug resistance genes can develop resistance to many different types of antibiotics [54], thereby facilitating broad bacterial distribution in diverse and changing environments [55]. Furthermore, VFGs were also reported in the environment [1,2]. Among the VFGs, adherence and invasion were represented with the highest abundance and two major types (offensive virulence factors and defensive virulence factors) were detected in HNA and LNA bacteria (Figure 1B,F), indicating that the VFG hosts in the samples have highly aggressive capability and lower resistance to the immunoreactions of humans/animals [56]. As reported in the previous study, ARGs and VFGs have strong correlation coefficients in bacteria [56,57], which was also found in the present study (Figure S4). Those results indicate the tendency of co-evolution between ARGs and VFGs in HNA and LNA bacteria [57,58]. VFGs are essential for bacteria to overcome host defense systems, and the acquirement of ARGs contributes to overcoming antimicrobial therapies and to adapting and colonizing demanding environments [59]. The coexistence fostered between VFGs and ARGs can spread across more bacterial genera and enhance the pathogenic potential within the environmental microbial community [60], when equipped with the proper transfer machinery and efficient vehicles for gene shuffling, such as mobile genetic elements (MGEs), including plasmids [61] and bacteriophages [62].

A previous study reported that environmental factors, such as salinity, nitrates, phosphates, and temperature, have an effect on ARG expression under global change in the oceans [3]. And environmental factor changes can promote the spread of VFGs and facilitate competition for nutrition and niches for survival [9]. Strikingly, the TPM values of total ARGs and VFGs in LNA bacteria showed an increasing trend with the decrease in salinity (Figure 1A,B). Under various salinity conditions, the TPM values of ARGs in the LNA bacterial community showed a decreasing trend with increasing salinity (*p* < 0.001) (Figure S5), including *efr*A, *sme*S, *tlr*C, *evg*S, *Nmc*R, *poxt*A (involved in multidrug resistance); *Tae*A (involved in pleuromutilin antibiotic); *bas*S, *ros*B, *bcr*A (involved in peptide antibiotic); *mac*B, *ole*C, *ole*B, *car*A (involved in macrolide antibiotic); *lmr*C (involved in lincosamide antibiotic); *pat*B and *pat*A (involved in fluoroquinolone antibiotic); *kdp*E (involved in aminoglycoside antibiotic); *nov*A (involved in aminocoumarin antibiotic). This could be explained by the phenomenon that the decrease in salinity leads to an increase in the diversity of LNA bacteria [23].

Environmental factors may indirectly shape ARG distribution by altering microbial community structures, as ARGs are closely linked to their microbial hosts [1]. The distribution of ARG hosts is one of the key determinants of ARG distribution patterns [52]. Environmental stresses such as extreme temperatures, pH, and salinity may induce bacteria to cope with these stresses through phenotypic and genotypic adaptations that enable subsequent resistance to similar stresses [63]. A remarkably strong consistency was found between the bacterial community composition and the composition of ARGs and VFGs in the different salinities in the present study (Figure 4). Further analysis revealed that both bacterial communities hosting ARGs and VFGs were affected by changes in salinity, with salinity explaining the greater variation in the LNA bacterial community composition hosting ARGs and VFGs (Tables S5 and S6).

The ecological and health risks of ARGs are mainly dependent on their microbial hosts, especially pathogenic bacteria, rather than the ARGs themselves [1]. The potential hosts of individual ARGs differ significantly in different habitats [53]. Previous studies reported that Proteobacteria, including Alphaproteobacteria and Gammaproteobacteria, is the major phyla commonly found in the marine environment [24,33,64], and they harbor ARGs or VFGs even when present in different environments [65,66,67]. Gammaproteobacteria, including the relevant families of *Enterobacteriaceae*, *Pseudomonadaceae*, and *Aeromonadaceae*, co-occur with the genes encoding beta-lactamase, sulfonamide, aminoglycoside, and tetracycline resistance [68]. *Pseudomonas* is notoriously known for the multidrug-resistance properties offered by its multidrug resistant efflux pump systems [69] and also carries a lot of virulence factor genes, such *tuf*A, *clp*V and *car*B [2], which was in line with the present study (Figure S9).

In the present study, we initially revealed the situation of ARGs carried by HNA and LNA bacteria in the marine environment, which has both advantages and disadvantages (Table S7). First, ARGs and VFGs can be transmitted between different microorganisms through horizontal gene transfer. Changes in salinity affect the interactions between microorganisms and the frequency of gene transfer, but currently, the specific mechanism by which salinity influences gene-level transfer is poorly understood, which limits the comprehensive understanding of the impact of salinity on the distribution of resistant genes and virulence factors. Second, future research will need to perform metagenomic assembly genome analysis to reveal the environmental microbial risks of HNA and LNA bacteria, and to explore their threats to human health.

## 5. Conclusions

In this study, we explored the effects of salinity fluctuation on ARGs and VFGs hosted by HNA and LNA bacteria in a marine environment. The principal findings of our study can be outlined as follows: the ARGs and VFGs hosted by HNA and LNA bacteria were significantly different under various salinity conditions in the marine environment. In addition, decreasing salinity significantly affected the ARGs’ and VFGs’ composition and the bacterial composition hosting the ARGs and VFGs. Further analysis showed that salinity is an important factor conditioning the bacterial composition of the aquatic environment hosting the ARGs and VFGs and further influences the quantity and distribution of ARGs and VFGs. Although more data are needed to evaluate the risks of the HNA and LNA bacterial hosts of ARGs and VFGs on environmental integrity in the marine environment, our aforementioned results emphasize the significant influence of salinity on the distribution of resistance genes and virulence factor genes in the marine environment.

## Data Availability

The original contributions presented in this study are included in the article/Supplementary Material. Further inquiries can be directed to the corresponding author.

## Supplementary Materials

The following supporting information can be downloaded at: https://www.mdpi.com/article/10.3390/microorganisms13071710/s1, Figure S1: Experimental design diagram; Figure S2: Schematic diagram of total bacterial concentration and the proportion of LNA and HNA bacteria at different salinities; Figure S3: The proportion of ARG types located at different genetic locations (A); ARG subtypes (top 50 most abundant) and associated resistance mechanisms (B). Typical arrangements of ARGs and MGEs in assembled contigs (C); Figure S4: Procrustes analysis showing the significant correlations between ARGs and VFGs; Figure S5: the correlations between ARGs and salinity; Figure S6: Co-occurrence networks depicting the interactions between VFGs and ARGs in both HNA and LNA bacteria. (A): the VFGs and ARGs in HNA bacteria; (B): the VFGs and ARGs in LNA bacteria; (C): the ARGs in both LNA and HNA bacteria; (D): the VFGs in both LNA and HNA bacteria; Figure S7: the community of the potential host HNA and LNA bacteria for ARGs and VFGs in different salinity at class level (Top 10); (A,C): the potential host HNA and LNA bacteria for ARGs; (B,D): the potential host HNA and LNA bacteria for VFGs; Figure S8: the community of the potential host HNA and LNA bacteria for ARGs and VFGs in different salinity at genus level (Top 10); (A,C): the potential host HNA and LNA bacteria for ARGs; (B,D): the potential host HNA and LNA bacteria for VFGs; Figure S9: ARGs and VFGs co-occurrence in PARB and principal coordinate analysis (PCoA) showing the distribution pattern of PARB in different salinities. (A,C): HNA bacteria; (B,D): LNA bacteria; Figure S10: Mantel analysis examining the relationships between HNA and LNA bacterial community and the abundance of ARGs (A) and VFGs (B); Figure S11: The correlations between the abundance of bacterial community and ARGs, VFGs. A, B: ARGs, C, D: VFGs; A, C: HNA bacteria; B, D: LNA bacteria. ***: *p* < 0.001, **: *p* < 0.01, *: *p* < 0.05; Table S1: Summary of environmental factors of all samples; Table S2: Partial mantel tests for the correlation between ARGs and environmental factors using Spearman’s coefficient; Table S3: Partial mantel tests for the correlation between VFGs and environmental factors using Spearman’s coefficient; Table S4: Topological parameters of the interaction networks of ARGs and VFGs in both HNA and LNA bacteria; Table S5: Partial mantel tests for the correlation between bacterial community hosting ARGs and environmental factors using Spearman’s coefficient; Table S6: Partial mantel tests for the correlation between bacterial community hosting VFGs and environmental factors using Spearman’s coefficient; Table S7: the advantages and advantages of the detection of ARGs and VFGs in HNA and LNA bacteria.
