# Supplementary material for: Effects of Salinity Fluctuation on Antimicrobial Resistance and Virulence Factor Genes of Low and High Nucleic Acid-Content Bacteria in a Marine Environment
Source: Microorganisms. 2025 Jul 21;13(7):1710. doi: 10.3390/microorganisms13071710 (PMC12298737; doi:10.3390/microorganisms13071710)
Supplement: Supplementary file 1 [file microorganisms-13-01710-s001.zip › microorganisms-3732271-supplementary.pdf]

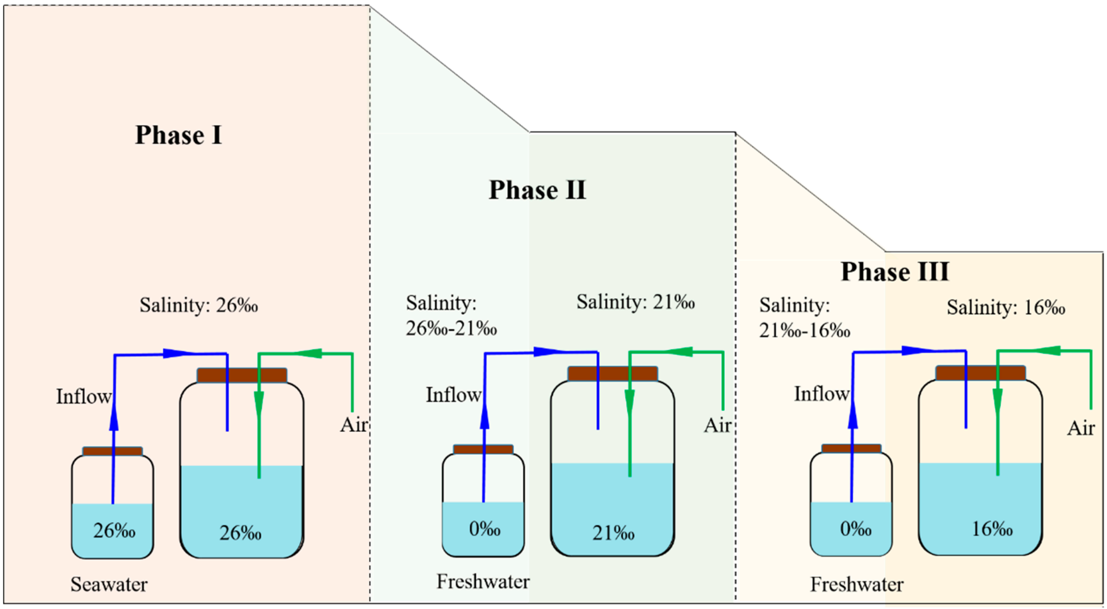

Figure S1 Experimental design diagram

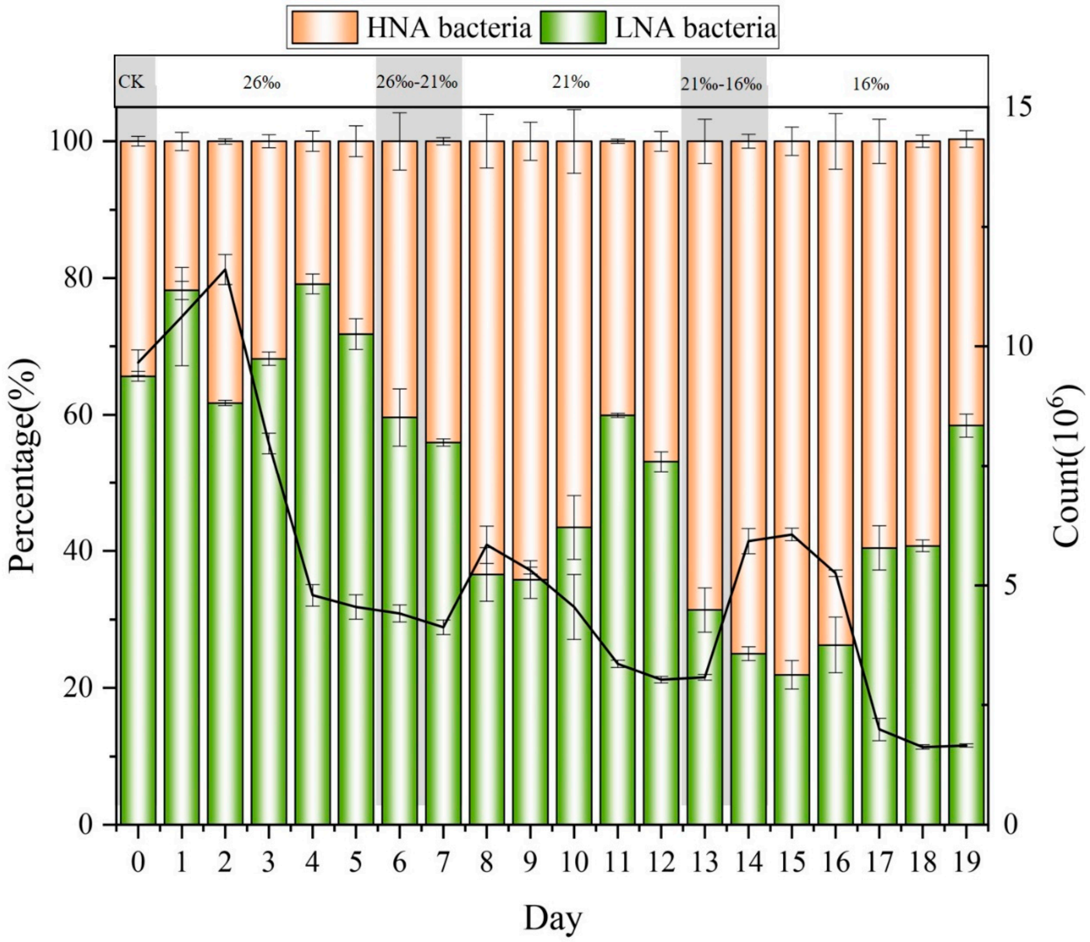

Figure S2 Schematic diagram of total bacterial concentration and the proportion of LNA and HNA bacteria at different salinities

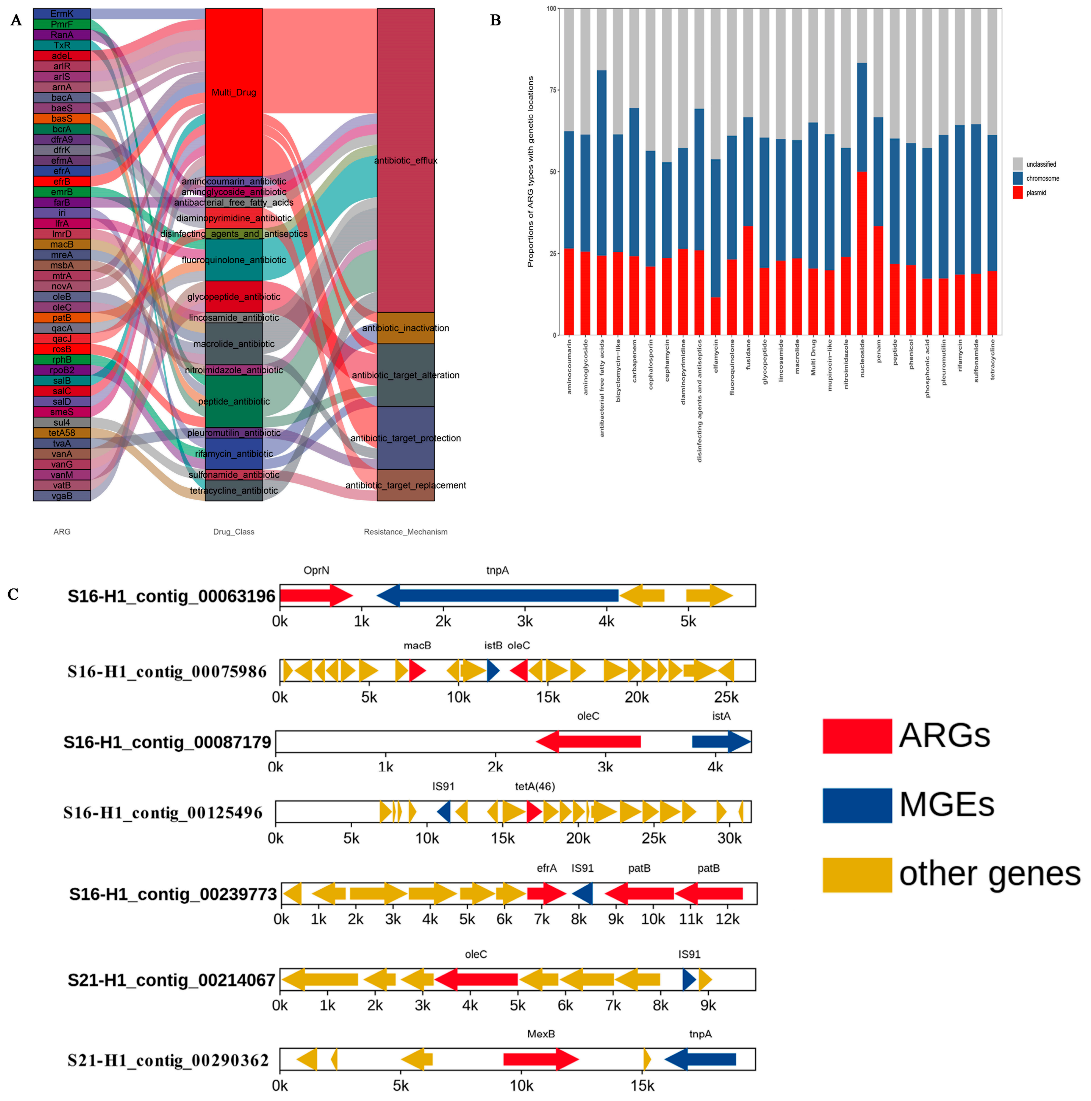

Figure S3 The proportion of ARG types located at different genetic locations (A); ARG subtypes (top 50 most abundant) and associated resistance mechanisms (B). Typical arrangements of ARGs and MGEs in assembled contigs (C)

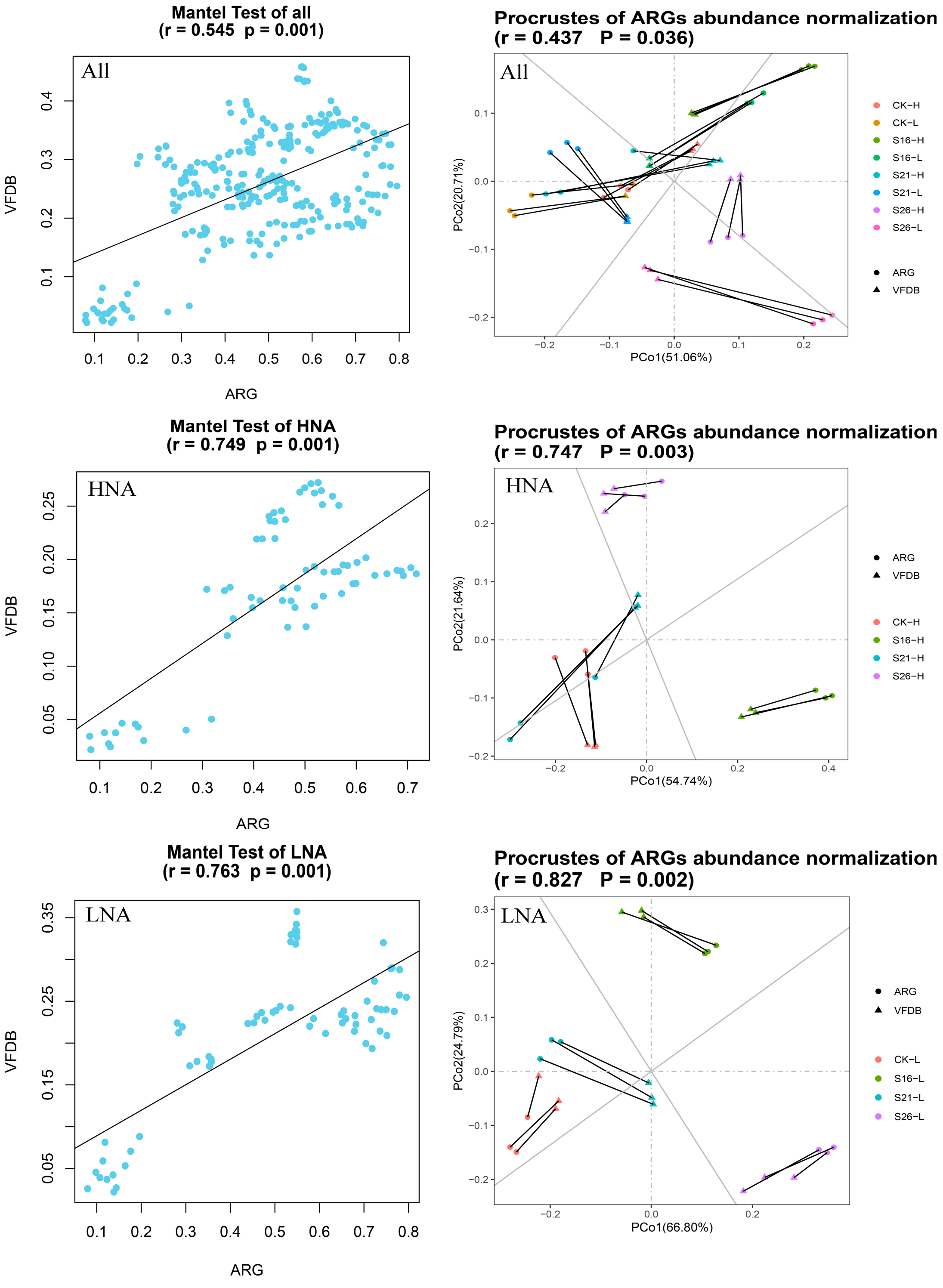

Figure S4 Procrustes analysis showing the significant correlations between ARGs and VFGs

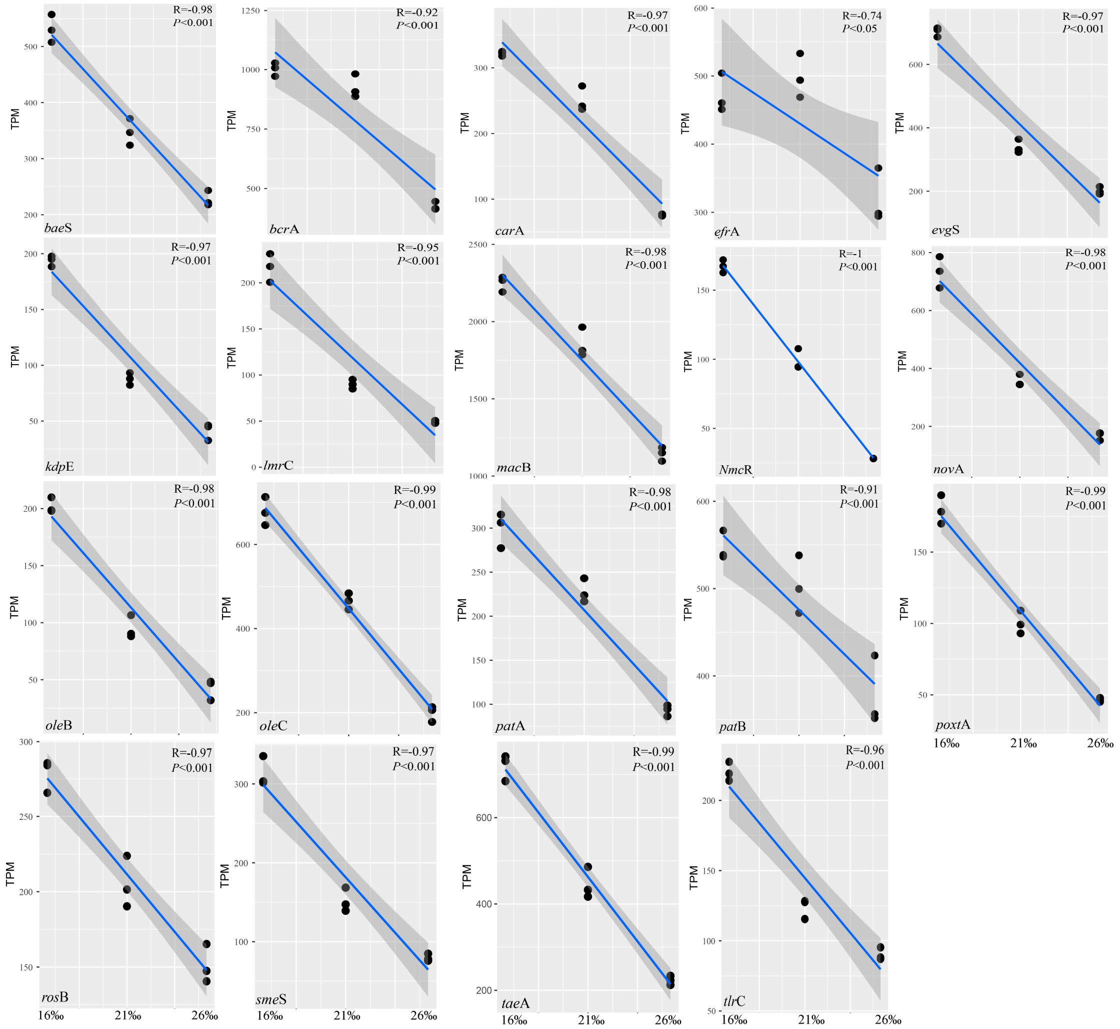

Figure S5 the correlations between ARGs and salinity

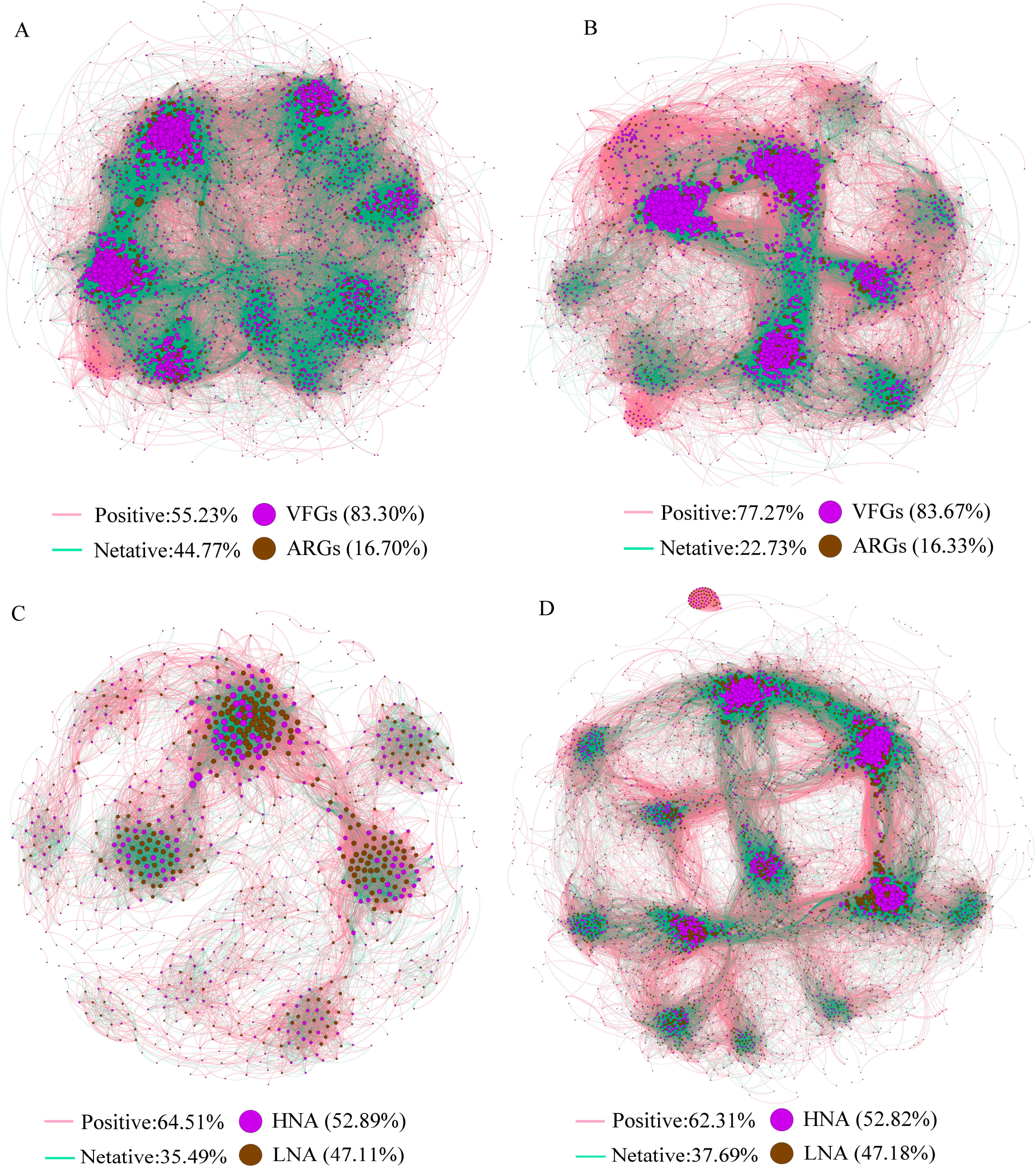

Figure S6 Co-occurrence networks depicting the interactions between VFGs and ARGs in both HNA and LNA bacteria. A: the VFGs and ARGs in HNA bacteria; B: the VFGs and ARGs in LNA bacteria; C: the ARGs in both LNA and HNA bacteria; D: the VFGs in both LNA and HNA bacteria

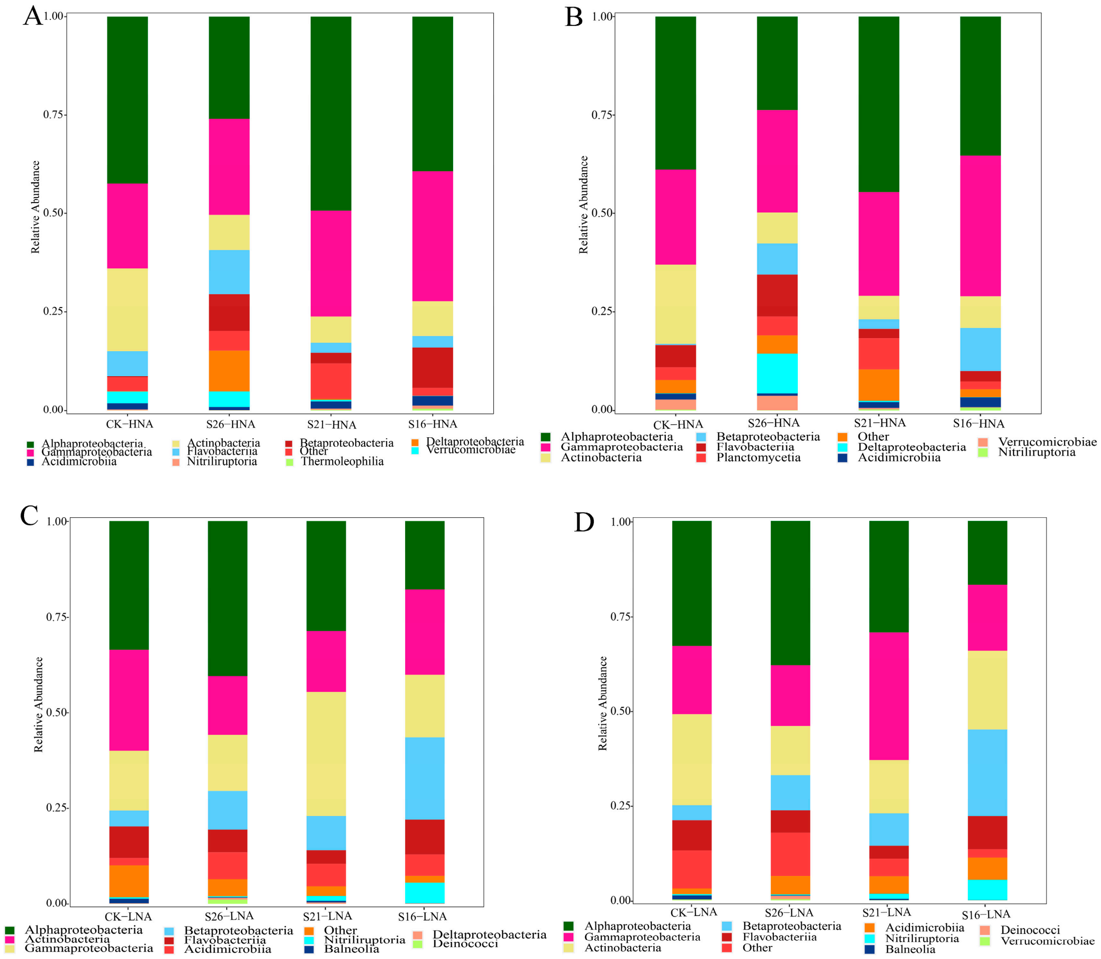

Figure S7 the community of the potential host HNA and LNA bacteria for ARGs and VFGs in different salinity at class level (Top 10); A,C: the potential host HNA and LNA bacteria for ARGs; B,D: the potential host HNA and LNA bacteria for VFGs

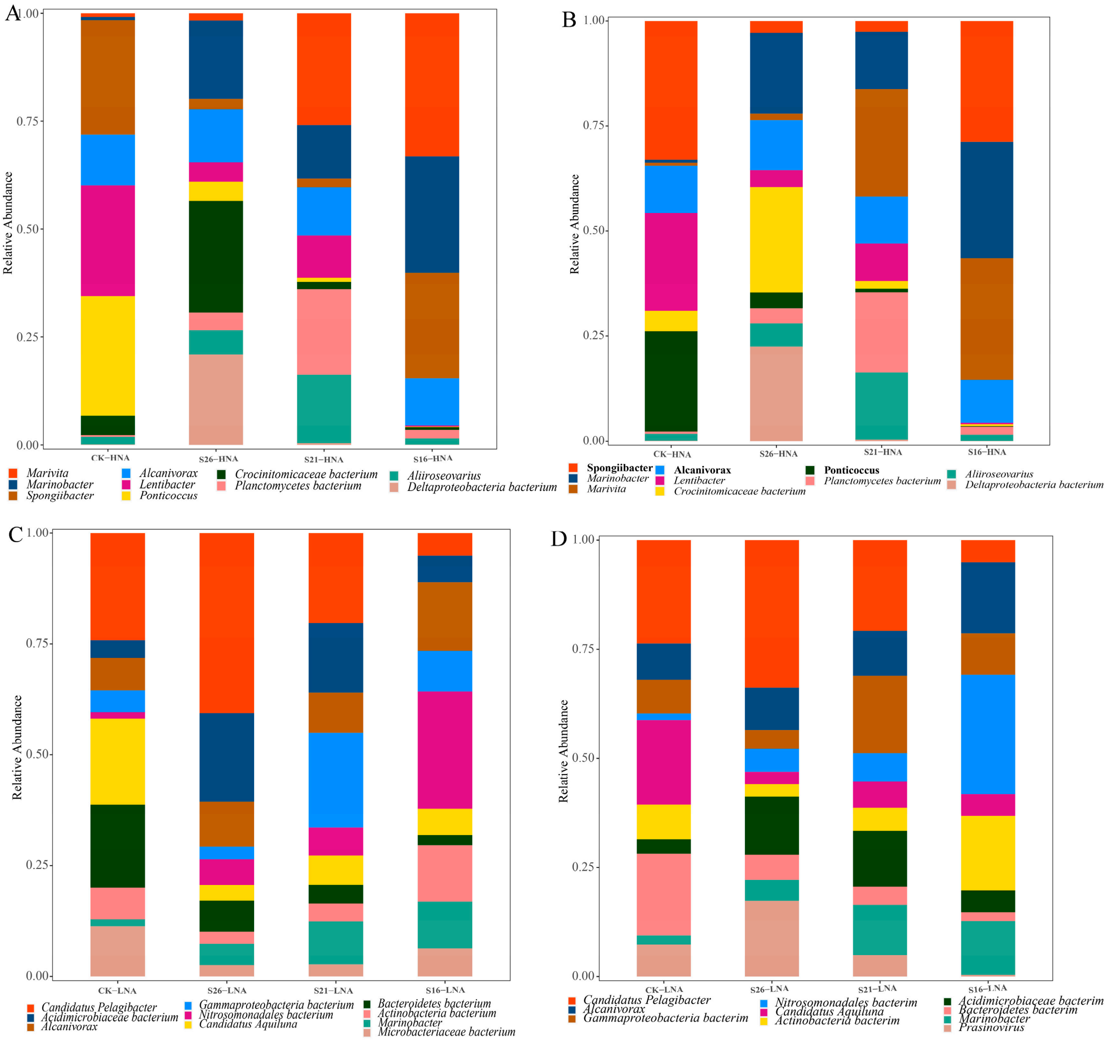

Figure S8 the community of the potential host HNA and LNA bacteria for ARGs and VFGs in different salinity at genus level (Top 10); A, C: the potential host HNA and LNA bacteria for ARGs; B, D: the potential host HNA and LNA bacteria for VFGs

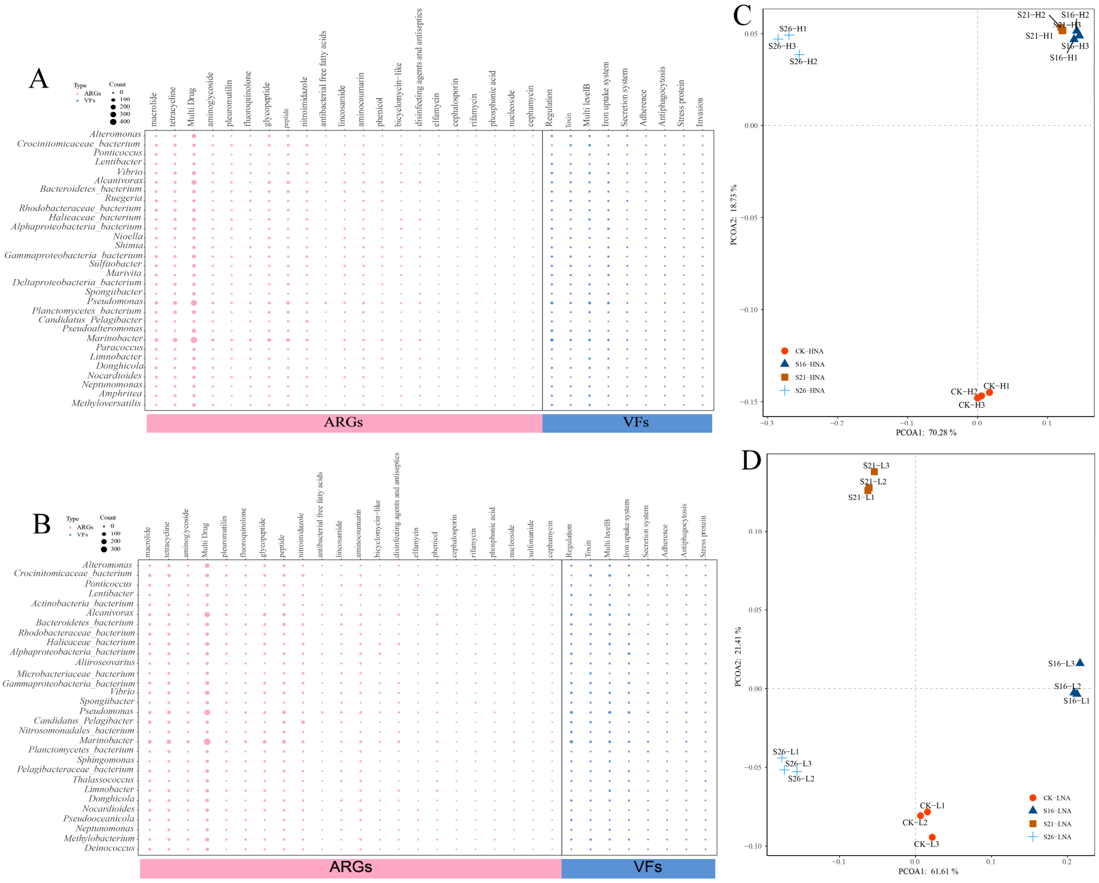

Figure S9 ARGs and VFGs co-occurrence in PARB and principal coordinate analysis (PCoA) showing the distribution pattern of PARB in different salinities. A, C: HNA bacteria; B, D: LNA bacteria.

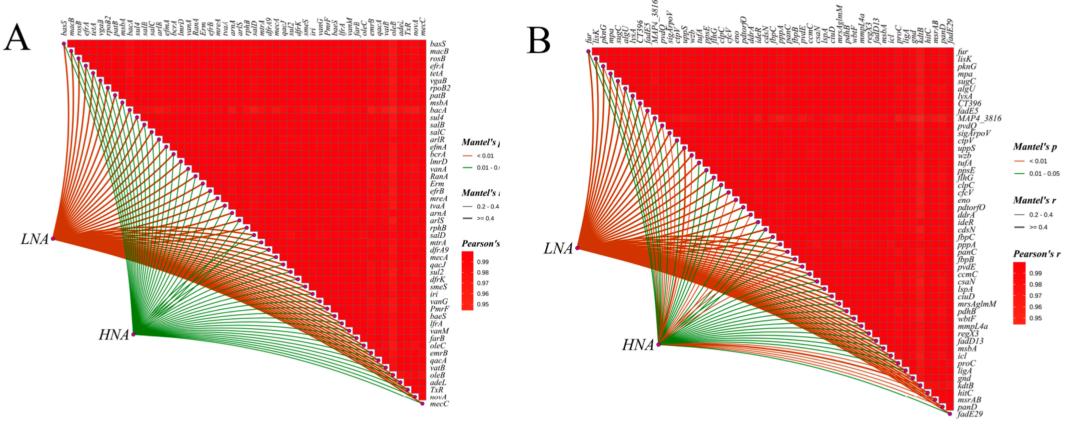

Figure S10 Mantel analysis examining the relationships between HNA and LNA bacterial community and the abundance of ARGs (A) and VFGs (B)

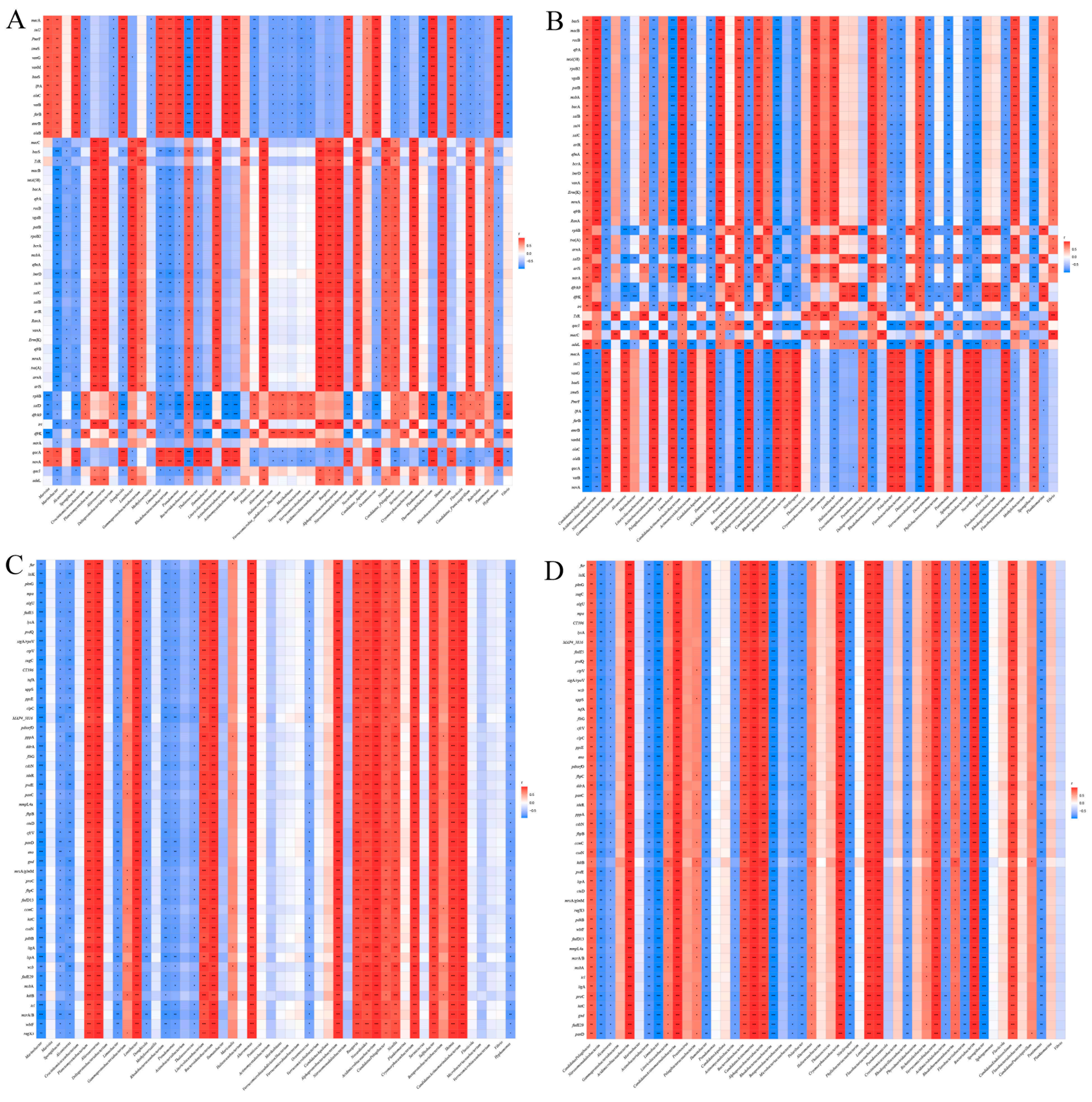

Figure S11 The correlations between the abundance of bacterial community and ARGs, VFGs. A, B: ARGs, C, D: VFGs; A, C: HNA bacteria; B, D: LNA bacteria. ***: *p*<0.001, **: *p*<0.01, *:*p*<0.05

Table S1 Summary of environmental factors of all samples

	T	Salinity	pH	DOC(mg/L)	DC(mg/L)	DN(mg/L)	DP(mg/L)
CK1	20.8	26	7.23	8.03	37.23	0.7344	0.11
CK2	20.7	26	7.23	8.37	37.55	0.7933	0.12
CK3	20.8	26	7.23	7.81	37.55	0.7055	0.11
S26-1	27.1	26	7.34	7.38	36.46	0.8269	0.11
S26-2	27.2	26	7.338	7.51	36.68	0.8096	0.12
S26-3	27.2	26	7.335	7.23	36.39	0.826	0.11
S21-1	27.5	21	7.476	9.13	38.14	0.8429	0.1
S21-2	27.4	22	7.487	8.1	38.6	0.72	0.09
S21-3	27.4	21	7.482	8.92	38.87	0.8462	0.1
S16-1	27.3	16	7.528	9.55	40.24	0.6419	0.1
S16-2	27.4	16	7.536	7.97	39.06	0.6847	0.09
S16-3	27.4	17	7.532	8.4	39.14	0.8147	0.1
Freshwater-1	27	0	8.09	13.71	49.99	0.4817	0.1
Freshwater-2	27	0	8.09	12.54	46.47	0.5256	0.11
Freshwater-3	27	0	8.09	12.03	47.16	0.5004	0.1

DOC: dissolved organic carbon; DC: Total dissolved carbon; DN: Total dissolved nitrogen; DP: total dissolved phosphorus

Table S2 Partial mantel tests for the correlation between ARGs and environmental factors using Spearman's coefficient

Effects of	Cotroll for	HNA bacteria		LNA bacteria	
		r-value	*p*-value	r-value	*p*-value
Salinity	T+pH+TC+TN+TOC+TP	0.717	0.001	0.776	0.014
TC	T+pH+salinity+TN+TOC+TP	0.4829	0.04	0.5743	0.003
T	pH+salinity+TC+TN+TOC+TP	0.2335	0.093	0.2148	0.126
pH	T+salinity+TC+TN+TOC+TP	0.7072	0.001	0.522	0.001
TOC	T+pH+salinity+TC+TN+TP	0.1334	0.018	0.0369	0.346
TN	T+pH+salinity+TC+TOC+TP	-0.1066	0.073	-0.4279	0.995
TP	T+pH+salinity+TC+TOC+TN	0.2402	0.08	0.1154	0.215

Table S3 Partial mantel tests for the correlation between VFGs and environmental factors using Spearman's coefficient

Effects of	Cotroll for	HNA bacteria		LNA bacteria	
		r-value	*p*-value	r-value	*p*-value
Salinity	T+pH+TC+TN+TOC+TP	0.5568	0.012	0.844	0.001
TC	T+pH+salinity+TN+TOC+TP	0.4996	0.006	0.645	0.001
T	pH+salinity+TC+TN+TOC+TP	0.1442	0.24	0.1636	0.186
pH	T+salinity+TC+TN+TOC+TP	0.82	0.001	0.5421	0.014
TOC	T+pH+salinity+TC+TN+TP	0.0882	0.247	0.0795	0.257
TN	T+pH+salinity+TC+TOC+TP	-0.3216	0.992	-0.3884	0.993
TP	T+pH+salinity+TC+TOC+TN	0.04536	0.336	0.1074	0.209

Table S4 Topological parameters of the interaction networks of ARGs and VFGs in both HNA and LNA bacteria

	HNA-ARGs-VFGs	LNA-ARGs-VFGs	HNA-LNA-ARGs	HNA-LNA-VFGs
node	2845	2523	864	4385
link	126,669	143,329	17080	229910
modularity	4.905	1.046	1.776	2.195

Table S5 Partial mantel tests for the correlation between bacterial community hosting ARGs and environmental factors using Spearman's coefficient

Effects of	Cotroll for	HNA bacteria		LNA bacteria	
		r-value	*p*-value	r-value	*p*-value
Salinity	T+pH+TC+TN+TOC+TP	0.5735	0.001	0.7934	0.003
TC	T+pH+salinity+TN+TOC+TP	0.2141	0.1	0.5838	0.002
T	pH+salinity+TC+TN+TOC+TP	-0.0366	0.47	-0.1466	0.801
pH	T+salinity+TC+TN+TOC+TP	0.428	0.012	0.56	0.002
TOC	T+pH+salinity+TC+TN+TP	0.2538	0.092	-0.0615	0.581
TN	T+pH+salinity+TC+TOC+TP	-0.4063	0.994	0.0514	0.339
TP	T+pH+salinity+TC+TOC+TN	0.0630	0.326	-0.1405	0.8

Table S6 Partial mantel tests for the correlation between bacterial community hosting VFGs and environmental factors using Spearman's coefficient

Effects of	Cotroll for	HNA bacteria		LNA bacteria	
		r-value	*p*-value	r-value	*p*-value
Salinity	T+pH+TC+TN+TOC+TP	0.0786	0.288	0.8732	0.002
TC	T+pH+salinity+TN+TOC+TP	0.3289	0.045	0.6217	0.002
T	pH+salinity+TC+TN+TOC+TP	0.5994	0.002	-0.2225	0.923
pH	T+salinity+TC+TN+TOC+TP	0.4861	0.008	0.5833	0.002
TOC	T+pH+salinity+TC+TN+TP	0.2463	0.08	-0.1012	0.783
TN	T+pH+salinity+TC+TOC+TP	-0.3956	0.999	0.0701	0.302
TP	T+pH+salinity+TC+TOC+TN	-0.0375	0.521	-0.208	0.938

Table S7 the advantages and advantages of the detection of ARGs and VFGs in HNA and LNA bacteria

Type	advantages	advantages
HNA bacteria	HNA bacteria means that the bacterial signal in the sample is stronger. When conducting genetic testing and analysis, it is easier to be accurately identified and quantified, and can more clearly reveal the distribution of ARGs and VFGs.	HNA bacteria often have an advantage in the environment and may mask the existence and distribution of ARGs and VFGs in LNA bacteria, resulting in an incomplete understanding of the diversity of bacterial ARGs and VFGs in the entire marine environment.
LNA bacteria	1. LNA bacteria in the marine environment may represent some rare or special ecological niches. Their existence and distribution may be closely related to specific environmental factors or ecological processes. Studying the distribution of ARGs and VFGs of these bacteria can provide a more comprehensive understanding of the bacterial community structure and function in the marine environment, and reveal some potential ecological mechanisms and environmental adaptation strategies.<br>2. LNA bacteria may possess unique ecological functions or metabolic pathways. Their ARGs and VFGs may be related to these special functions. Studying their distribution can provide new perspectives and clues for exploring the material cycling, energy flow and biogeochemical processes of marine ecosystems.	1.Due to the low nucleic acids, when conducting genetic testing and analysis, it is prone to be affected by background noise and contamination, resulting in a decrease in the accuracy and reliability of the test results.<br>2. In analyses such as metagenomic sequencing, due to the low nucleic acid content, more water samples need to be collected.
